# Analytical treatment of proton double-quantum NMR intensity buildup: multi-spin couplings and the flip-flop term

**DOI:** 10.5194/mr-6-1-2025

**Published:** 2025-01-14

**Authors:** Nail Fatkullin, Ivan Brekotkin, Kay Saalwächter

**Affiliations:** 1 Institut für Physik – NMR, Martin-Luther-Universität Halle-Wittenberg, 06120 Halle (Saale), Germany; 2 Institute of Physics, Kazan Federal University, 420008 Kazan, Tatarstan, Russia

## Abstract

A modified Anderson–Weiss approximation for describing double-quantum (DQ) NMR experiments in systems with many I = 1/2 spins is proposed, taking inter-spin flip-flop processes into special consideration. In this way, an analytical result is derived for multi-spin systems for the first time. It is shown that in the initial stages of DQ intensity buildup, the probability of flip-flop processes in DQ experiments is half as large as in analogous Hahn echo or free induction decay experiments. Their influence on the experimentally observed DQ NMR signal becomes dominant at times $t>{\left(9/2\right)}^{1/2}T_{2}^{\mathrm{eff}}\approx 2.12T_{2}^{\mathrm{eff}}$ , where $T_{2}^{\mathrm{eff}}$ is the effective spin–spin relaxation time measured by the Hahn echo. Calculations and a comparison with spin dynamics simulations of small spin systems of up to eight spins reveal a satisfactory agreement.

## 1 Introduction

The seminal papers by Baum and Pines (Baum et al., 1985; Baum and Pines, 1986) started the field of double-quantum (DQ) or, more generally, multiple-quantum (MQ) NMR. At the qualitative level, the idea of the method is quite simple. The system of spins under study is continuously irradiated by a special sequence of radiofrequency (RF) pulses, which allows one to change the relative importance of different parts of the initial Hamiltonian of spin–lattice interactions, inducing different quantum transitions in the spin system. In other words, the irradiation pattern creates a new effective interaction Hamiltonian that induces selective quantum transitions in the spin system. In the mentioned initial papers (Baum et al., 1985; Baum and Pines, 1986), the method was mathematically justified for solids in which thermal motions of spins can be neglected in comparison with the initial spatial distances between them. An additional feature of solids is the large difference between the spin–spin and spin–lattice relaxation time, $T_{2}^{\mathrm{eff}}\ll T_{1}$ . This allows us, at each time much shorter than the spin–lattice relaxation time, $t\ll T_{1}$ , to neglect the influence of the so-called non-secular part of the spin–lattice interaction Hamiltonian on the dynamics of the spins under study and to limit ourselves to considering only its secular part.

Subsequently (see, for example, Graf, 1998; Dollase et al., 2001; Saalwächter, 2002a, b, 2007; Saalwächter et al., 2003, 2006; Saalwächter and Heuer, 2006; Fechete et al., 2002; Mordvinkin and Saalwächter, 2017; Mordvinkin et al., 2020; Vaca Chavez and Saalwächter, 2011; Shahsavan et al., 2022, and references therein), the method was phenomenologically generalized for the case when the relative spatial displacements of spins during the experiment cannot be considered small, but the situation is still such that $T_{2}^{\mathrm{eff}}\ll T_{1}$ . In recent work (Brekotkin et al., 2022), it has been proven by methods of statistical physics that in the limit Δ→0 , where Δ is the time interval between the nearest RF pulses, the phenomenological method of accounting for spatial displacements of spins during the experiment gives correct results. Thus, a general relation allowing for a quantitative account of the corresponding corrections was obtained. Relevant analytical results related to DQ NMR have recently been obtained for model solid-state many-spin one-dimensional I = 1/2 systems in which magnetic dipole–dipole interactions have been considered only between nearest neighbors (see Bochkin et al., 2022; Bochkin et al., 2024; Fel'dman et al., 2024; Doronin et al., 2000, and references cited therein).

For the case of the spin system I = 1/2 , the dominant interactions determining the spin relaxation are, as a rule, magnetic dipole–dipole interactions. The secular part of the Hamiltonian has the following form (see, for example, Fatkullin et al., 2012; Fatkullin et al., 2013):

1 $$\begin{array}{rl} {\hat{H}}_{dd}^{\sec} & =\sum_{i<j}\hbar \omega_{ij}\left(2{\hat{I}}_{i}^{z}{\hat{I}}_{j}^{z}-{\hat{I}}_{i}^{x}{\hat{I}}_{j}^{x}-{\hat{I}}_{i}^{y}{\hat{I}}_{j}^{y}\right)\\ & =\sum_{i<j}\hbar \omega_{ij}\left(2{\hat{I}}_{i}^{z}{\hat{I}}_{j}^{z}-\frac{1}{2}\left({\hat{I}}_{i}^{+}{\hat{I}}_{j}^{-}+{\hat{I}}_{i}^{-}{\hat{I}}_{j}^{+}\right)\right), \end{array}$$

where ${\hat{I}}_{k}^{+}={\hat{I}}_{k}^{x}+i{\hat{I}}_{k}^{y}$ , ${\hat{I}}_{k}^{+}={\hat{I}}_{k}^{x}+i{\hat{I}}_{k}^{y}{\hat{I}}_{k}^{-}={\hat{I}}_{k}^{x}-i{\hat{I}}_{k}^{y}$ . The parameter $\omega_{ij}$ describes in frequency units the effective strength of the dipole–dipole coupling of spins with numbers i and j . It is given by the following expression:

2 $$\omega_{ij}=\frac{1}{8\pi }\frac{\mu_{0}\gamma^{2}\hbar \left(1-3{\mathrm{cos}}^{2}\theta_{ij}\right)}{r_{ij}^{3}}=D^{ij}\frac{\left(1-3{\mathrm{cos}}^{2}\theta_{ij}\right)}{2},$$

where $r_{ij}$ is the distance between interacting spins; $\theta_{ij}$ is the angle between direction z , defined as the direction along which the external magnetic field is aligned, and the vector connecting the discussed spins; ${\hat{I}}_{i}^{\alpha }$ is the operator of the α component of the spin with number i ; ℏ is Planck's constant divided by 2π ; $\mu_{0}$ is the magnetic field constant; and γ is the gyromagnetic ratio of the spins. $D^{ij}=\mu_{0}\gamma^{2}\hbar /\left(4\pi r_{ij}^{3}\right)$ is the dipole–dipole coupling constant. The Hamiltonian (Eq. 1) in this paper plays the role of the original spin–lattice interaction Hamiltonian. In the lowest order of perturbation theory, it induces in a spin system only zero-quantum transitions (dephasing processes or zero-quantum coherence) through terms proportional to ${\hat{I}}_{i}^{z}{\hat{I}}_{j}^{z}$ and flip-flop processes proportional to ${\hat{I}}_{i}^{+}{\hat{I}}_{j}^{-}+{\hat{I}}_{i}^{-}{\hat{I}}_{j}^{+}$ . We also note that the non-secular part of the Hamiltonian of magnetic dipole–dipole interactions inducing the processes of spin–lattice relaxation describes the processes of single-quantum and double-quantum coherence. However, at each time of interest, $t\ll T_{1}$ , they can be neglected.

Under irradiation of the spin system by a special RF pulse sequence referred to as the Baum–Pines (BP) sequence (see details in Baum et al., 1985; Baum and Pines, 1986), the dynamics of the spin system at each time $t\ll T_{1}$ is determined by not only the Hamiltonian Eq. (1) but also the effects associated with the irradiation. In fact, there are two conceptually different experiments, each consisting of two stages of equal duration $\tau_{\mathrm{DQ}}$ . The first half of both experiments is called the excitation stage, and the second half is called the reconversion stage. At the moment of time $2\tau_{\mathrm{DQ}}$ the signal of the studied spin system is measured. Normally, a four-step phase cycle is applied to the relative overall phase of the reconversion stage in combination with the receiver phase to filter for either (4 n+2 )-quantum coherences (“DQ signal”) or 4 n -quantum coherences (“reference signal”). In the following, we use a simplified yet equivalent description of these two experiments (Saalwächter, 2013). They essentially differ from each other in the fact that in the first case, during both periods of the experiment, the phase of RF exposure does not change, and in the second experiment, during the period of reconversion, the phase of RF exposure changes by 90°, which changes a sign of the of the resulting effective spin Hamiltonian, i.e., performs the time-reversal operation with respect to the spin variables. We denote the measured signal in the first experiment by $A_{0}\left(2\tau_{\mathrm{DQ}}\right)$ and in the second experiment by $A_{1}\left(2\tau_{\mathrm{DQ}}\right)$ .

In solids, the joint effect of the BP sequence and the Hamiltonian Eq. (1), in the limit Δ→0 , where Δ is the time interval between the nearest RF pulses, the spin system's time evolution can be described in terms of an effective DQ Hamiltonian with the following structure:

3 $$\begin{array}{rl} {\hat{H}}_{\mathrm{DQ}}^{n} & ={\left(-1\right)}^{n\theta \left(t-\tau_{\mathrm{DQ}}\right)}\sum_{i<j}\hbar \omega_{ij}\left({\hat{I}}_{i}^{y}{\hat{I}}_{j}^{y}-{\hat{I}}_{i}^{x}{\hat{I}}_{j}^{x}\right)\\ & ={\left(-1\right)}^{n\theta \left(t-\tau_{\mathrm{DQ}}\right)}\sum_{i<j}\frac{\hbar \omega_{ij}}{2}\left({\hat{I}}_{i}^{+}{\hat{I}}_{j}^{+}+{\hat{I}}_{i}^{-}{\hat{I}}_{j}^{-}\right), \end{array}$$

where $\theta \left(x\right)$ is the Heaviside step function, n=0,1 . The case n=0 corresponds to the first mentioned version of the DQ experiment without phase change, i.e., to the signal $A_{0}\left(2\tau_{\mathrm{DQ}}\right)$ , and n=1 to the second version with a 90° phase shift, i.e., to the signal $A_{1}\left(2\tau_{\mathrm{DQ}}\right)$ . Note also that the Hamiltonian Eq. (3) for the case of solids is usually derived by the average Hamiltonian theory (see, e.g., Haeberlen, 1976) and can, in principle, also be obtained also via the Floquet formalism (see, e.g., Ivanov et al., 2021, and references cited therein).

In the lowest order of perturbation theory, or each time $t<T_{\mathrm{fl}}^{*}$ , where $T_{\mathrm{fl}}^{*}$ is the characteristic time of flip-flop processes created by the Hamiltonian Eq. (1), the Hamiltonian Eq. (3), in contrast to Eq. (1), induces double-quantum (DQ) transitions (or creates DQ coherences) of interacting spins. At longer times, of course, more complex quantum transitions involving coherent behavior of an even number of spins become essential. At a time $t=\tau_{\mathrm{DQ}}$ , the operator in Eq. (3), changes its sign, which is dynamically equivalent to a time-reversal operation for the case when n=1 .

For situations where spins are moving, Eq. (3) was heuristically generalized by considering the coupling constant as a function of time. The validity of such a generalization is shown by Brekotkin et al. (2022) by a sequential quantum statistical calculation showing that the terms added to the relation vanish in the limit Δ→0 ; in addition, a general relation is obtained that allows one to quantify the corresponding contributions to the experimentally measured signal, if necessary.

In this paper, we ignore the mentioned effect and work with an effective Hamiltonian of the form

4 $${\hat{H}}_{\mathrm{DQ}}^{n}\left(t\right)=\sum_{i<j}\hbar \omega_{ij}^{\left(n\right)}\left(t\right)\left({\hat{I}}_{i}^{y}{\hat{I}}_{j}^{y}-{\hat{I}}_{i}^{x}{\hat{I}}_{j}^{x}\right),$$

where

5 $$\omega_{ij}^{\left(n\right)}\left(t\right)={\left(-1\right)}^{n\theta \left(t-\tau_{\mathrm{DQ}}\right)}\omega_{ij}\left(t\right).$$

Here, $\omega_{ij}\left(t\right)$ can be obtained from $\omega_{ij}$ of Eqs. (1) and (3) by transition to the ordinary quantum mechanical interaction (Dirac) representation, where the role of the zeroth-order Hamiltonian, i.e., the zero-point Hamiltonian or the dominant part of the Hamiltonian (see also Eq. 8), is a sum of the lattice Hamiltonian and the Zeeman Hamiltonian of the investigated spins with an external magnetic field. Recall that the properties of the Heaviside step function used in Eq. (5) are such that $\theta \left(x\right)=0$ at x<0 and $\theta \left(x\right)=1$ at x≥0 , which allows us to analytically account for the inverted sign of the DQ Hamiltonian during the reconversion period in experiments when n=1 .

The Hamiltonian Eq. (4) itself is already quite complex and does not allow for accurate calculations in nontrivial cases. The standard approximation that allows one to obtain closed analytic relations is the Anderson–Weiss (AW) approximation (Anderson and Weiss, 1953; Abragam, 1981; Kimmich, 1997), the second cumulant approximation in common parlance, which completely ignores the effects of flip-flop processes. Alternatively, the Hamiltonian Eq. (4) can be rewritten in the following form:

6 $$\begin{array}{rl} {\hat{H}}_{\mathrm{DQ}}^{\left(n\right)}\left(t\right) & =\sum_{i<j}2\hbar \omega_{ij}^{\left(n\right)}\left(t\right){\hat{I}}_{i}^{y}{\hat{I}}_{j}^{y}\\ & -\sum_{i<j}\hbar \omega_{ij}^{\left(n\right)}\left(t\right)\left({\hat{I}}_{i}^{y}{\hat{I}}_{j}^{y}+{\hat{I}}_{i}^{x}{\hat{I}}_{j}^{x}\right)\\ & =\sum_{i<j}2\hbar \omega_{ij}^{\left(n\right)}\left(t\right){\hat{I}}_{i}^{y}{\hat{I}}_{j}^{y}\\ & -\frac{1}{2}\sum_{i<j}\hbar \omega_{ij}^{\left(n\right)}\left(t\right)\left({\hat{I}}_{i}^{+}{\hat{I}}_{j}^{-}+{\hat{I}}_{i}^{-}{\hat{I}}_{j}^{+}\right). \end{array}$$

By direct calculation one can see that

7 $$\begin{array}{rl} & \left[{\hat{I}}^{z};\sum_{i<j}\hbar \omega_{ij}^{\left(n\right)}\left(t\right)\left({\hat{I}}_{i}^{y}{\hat{I}}_{j}^{y}+{\hat{I}}_{i}^{x}{\hat{I}}_{j}^{x}\right)\right]\\ & \;\;=\frac{1}{2}\sum_{i<j}\hbar \omega_{ij}^{\left(n\right)}\left(t\right)\left[{\hat{I}}_{z};{\hat{I}}_{i}^{+}{\hat{I}}_{j}^{-}+{\hat{I}}_{i}^{-}{\hat{I}}_{j}^{+}\right]=0, \end{array}$$

where ${\hat{I}}^{z}=\sum_{k}{\hat{I}}_{k}^{z}$ is the z component of the total spin system.

This exact result allows us to modify the usual Anderson–Weiss approximation so that the effects of flip-flop processes on the experimentally observed signals will be accounted for at least in the mean-field approximation. A detailed study of this circumstance is the main purpose of this article.

## 2 Theoretical part

### 2.1 General considerations

An inevitable and important initial element of all quantum statistical calculations of experimentally measured dynamical quantities is the transition to the interaction representation, synonymous to the Dirac representation. This transition is performed by dividing the full initial Hamiltonian of the system, $\hat{H}$ , into a sum of the zeroth-order Hamiltonian, ${\hat{H}}_{0}$ , and the “interaction” Hamiltonian, ${\hat{H}}_{\mathrm{int}}$ :

8 $$\hat{H}={\hat{H}}_{0}+{\hat{H}}_{\mathrm{int}}.$$

In problems of NMR spectroscopy, the lattice Hamiltonian, ${\hat{H}}_{L}$ , and the Hamiltonian of the Zeeman interaction of the spins under study ( ${\hat{H}}_{Z}=\hbar \omega_{0}{\hat{I}}_{z}$ , $\omega_{0}$ being the resonance frequency), are usually included in ${\hat{H}}_{0}={\hat{H}}_{L}+{\hat{H}}_{Z}$ ; all other interactions are assumed to be included in ${\hat{H}}_{\mathrm{int}}$ . In the DQ experiments discussed in this paper, the measured quantity is the z component of the total system spin ${\hat{I}}^{z}=\sum_{k}{\hat{I}}_{k}^{z}$ . The existence of the identity Eq. (7) allows one to include the DQ part of the Hamiltonian

9 $${\hat{H}}_{\mathrm{DQ}}^{\mathrm{fl}}\equiv -\sum_{i<j}\hbar \omega_{ij}^{\left(n\right)}\left(t\right)\left({\hat{I}}_{i}^{y}{\hat{I}}_{j}^{y}+{\hat{I}}_{i}^{x}{\hat{I}}_{j}^{x}\right)$$

in the zeroth-order part of the Hamiltonian, thereby reformulating the transition into the Dirac representation. Only such a procedure should be accurately described since the initial Hamiltonian itself, Eq. (3), is an effective Hamiltonian generated by the joint action on the spin system of the secular part of the Hamiltonian of magnetic dipole–dipole interactions, Eq. (1), and the irradiation of the RF spin system by the Baum–Pines sequence.

In DQ NMR experiments described in the laboratory coordinate system and the Schrödinger representation, based upon irradiation of the spin system of the BP sequence (or its equivalent modifications), we have an initially time-dependent Hamiltonian

10 $${\hat{H}}^{\left(n\right)}\left(t\right)={\hat{H}}_{L}+{\hat{H}}_{Z}+{\hat{H}}_{dd}^{\sec}+{\hat{H}}_{\mathrm{BP}}^{\left(n\right)}\left(t\right),$$

where ${\hat{H}}_{\mathrm{BP}}^{\left(n\right)}\left(t\right)$ is the Hamiltonian of the interaction of the studied spin system with the irradiation field of the BP pulse sequence of type n=0,1 . Consequently, the evolution operator is initially a Dyson chronological, time-ordered, exponential propagator:

11 $${\hat{U}}^{\left(\mathrm{DQ},n\right)}\left(t\right)=\hat{T}\mathrm{exp}\left\{-\frac{i}{\hbar }\int_{0}^{t}{\hat{H}}^{\left(n\right)}\left(t_{1}\right)dt_{1}\right\}.$$

The transition into the interaction representation will be carried out in two steps. First, the following Hamiltonian plays the role of the zeroth-order Hamiltonian as usual:

12 $${\hat{H}}_{0}\left(t\right)={\hat{H}}_{L}+{\hat{H}}_{Z}.$$

The Eq. (11) can then be rewritten as follows:

13 $${\hat{U}}^{\left(\mathrm{DQ},n\right)}\left(t\right)={\hat{U}}_{0}\left(t\right){\hat{U}}_{1}^{\left(\mathrm{DQ},n\right)}\left(t\right),$$

where

14 $$\begin{array}{rl} & {\hat{U}}_{0}\left(t\right)=\hat{T}\mathrm{exp}\left\{-\frac{i}{\hbar }\int_{0}^{t}{\hat{H}}_{0}dt_{1}\right\}=\mathrm{exp}\left\{-\frac{i}{\hbar }{\hat{H}}_{0}t\right\},\\ & {\hat{U}}_{1}^{\left(\mathrm{DQ},n\right)}\left(t\right)=\hat{T}\mathrm{exp}\left\{-\frac{i}{\hbar }\int_{0}^{t}\left({\hat{H}}_{dd}^{\sec\left(n\right)}\left(t_{2}\right)+{\hat{\tilde{H}}}_{\mathrm{BP}}^{\left(n\right)}\left(t_{2}\right)\right)dt_{2}\right\},\\ & {\hat{H}}_{dd}^{\sec\left(n\right)}\left(t_{2}\right)+{\hat{\tilde{H}}}_{\mathrm{BP}}^{\left(n\right)}\left(t_{2}\right)={\left({\hat{U}}_{0}^{\left(\mathrm{DQ},n\right)}\left(t_{2}\right)\right)}^{-1}\\ & \;\;\;\left({\hat{H}}_{dd}^{\sec}+{\hat{H}}_{\mathrm{BP}}^{\left(n\right)}\left(t_{2}\right)\right){\hat{U}}_{0}^{\left(\mathrm{DQ},n\right)}\left(t_{2}\right). \end{array}$$

In the limit Δ→0 , according to Brekotkin et al. (2022), we have

15 $$\begin{array}{rl} {\hat{H}}_{dd}^{\sec\left(n\right)}\left(t_{2}\right) & +{\hat{\tilde{H}}}_{\mathrm{BP}}^{\left(n\right)}\left(t_{2}\right)\overset{\Delta \to 0}{⟶}{\hat{H}}_{\mathrm{DQ}}^{\left(n\right)}\left(t_{2}\right)\\ & =\sum_{i<j}\hbar \omega_{ij}^{\left(n\right)}\left(t_{2}\right)\left({\hat{I}}_{i}^{y}{\hat{I}}_{j}^{y}-{\hat{I}}_{i}^{x}{\hat{I}}_{j}^{x}\right). \end{array}$$

Now we can return to Eq. (6) and introduce the following notations to shorten the formulae:

16 $${\hat{H}}_{{}^{\prime }DQ}^{\left(n\right)}\left(t\right)={\hat{H}}_{\mathrm{DQ},yy}^{\left(n\right)}\left(t\right)+{\hat{H}}_{\mathrm{DQ},\mathrm{fl}}^{\left(n\right)}\left(t\right),$$

where

17 $${\hat{H}}_{\mathrm{DQ},yy}^{\left(n\right)}\left(t\right)=\sum_{i<j}2\hbar \omega_{ij}^{\left(n\right)}\left(t\right){\hat{I}}_{i}^{y}{\hat{I}}_{j}^{y}$$

and

18 $${\hat{H}}_{\mathrm{DQ},\mathrm{fl}}^{\left(n\right)}\left(t\right)=-\sum_{i<j}\hbar \omega_{ij}^{\left(n\right)}\left(t\right)\left({\hat{I}}_{i}^{y}{\hat{I}}_{j}^{y}+{\hat{I}}_{i}^{x}{\hat{I}}_{j}^{x}\right).$$

This represents the key step of our new approach as the Hamiltonian Eq. (18) commutes with the Zeeman Hamiltonian and can thus be absorbed in a new zero-point Hamiltonian to be used for the transition to another interaction frame, as discussed below.

In DQ experiments, the measured quantity is the z component of the total magnetic moment of the resonant spins, which in turn is proportional to the z component of the total spin ${\hat{I}}^{z}=\sum_{k}{\hat{I}}_{k}^{z}$ . The measurement, as already noted, is carried out for a time $t=2\tau_{\mathrm{DQ}}$ . In the high-temperature approximation by spin variables, the signal with n=0,1 for a given spin system is

19 $$\begin{array}{rl} & A_{n}\left(2\tau_{\mathrm{DQ}}\right)=\frac{\beta \hbar \omega_{0}}{{\left(2I+1\right)}^{N_{s}}}\\ & \;\;{\langle {\mathrm{Tr}}_{s}\left({\hat{I}}^{z}{\hat{U}}^{\left(\mathrm{DQ},n\right)}\left(2\tau_{\mathrm{DQ}}\right){\hat{I}}^{z}{\hat{U}}^{*\left(\mathrm{DQ},n\right)}\left(2\tau_{\mathrm{DQ}}\right)\right)\rangle }_{\mathrm{eq}}, \end{array}$$

where $N_{s}$ is the total number of spins in the system with the resonance frequency, $\omega_{0}$ ; β is the inverse temperature; and the trace operation, Tr ${}_{s}\left(\ldots \right)$ , is performed over the spin variables. Due to the unitarity of the propagator, one has ${\hat{U}}^{*\left(\mathrm{DQ},n\right)}\left(t\right)={\left({\hat{U}}^{\left(\mathrm{DQ},n\right)}\left(t\right)\right)}^{-1}$ , and the bracket ${\langle \ldots \rangle }_{\mathrm{eq}}$ denotes equilibrium averaging over all lattice variables.

Note once again that $\left[{\hat{I}}^{z};{\hat{H}}_{0}\right]=0$ , which allows us to rewrite Eq. (19) as follows:

20 $$\begin{array}{rl} & A_{n}\left(2\tau_{\mathrm{DQ}}\right)=\frac{\beta \hbar \omega_{0}}{{\left(2I+1\right)}^{N_{s}}}\\ & \;\;{\langle {\mathrm{Tr}}_{s}\left({\hat{I}}^{z}{\hat{U}}_{1}^{\left(\mathrm{DQ},n\right)}\left(2\tau_{\mathrm{DQ}}\right){\hat{I}}^{z}{\hat{U}}_{1}^{*(DQ.n)}\left(2\tau_{\mathrm{DQ}}\right)\right)\rangle }_{\mathrm{eq}}. \end{array}$$

Next, we represent the propagator ${\hat{U}}_{1}^{\left(\mathrm{DQ},n\right)}\left(t\right)$ as follows:

21 $$\begin{array}{rl} {\hat{U}}_{1}^{\left(\mathrm{DQ},n\right)}\left(t\right) & =\left(\hat{T}\mathrm{exp}\left(-\frac{i}{\hbar }\int_{0}^{t}{\hat{H}}_{\mathrm{DQ},\mathrm{fl}}^{\left(n\right)}\left(t_{1}\right)dt_{1}\right)\right)\\ & \left(\hat{T}\mathrm{exp}\left(-\frac{i}{\hbar }\int_{0}^{t}{\hat{\tilde{H}}}_{\mathrm{DQ},yy}^{\left(n\right)}\left(t_{1}\right)dt_{1}\right)\right), \end{array}$$

where

22 $$\begin{array}{rl} {\hat{\tilde{H}}}_{\mathrm{DQ},yy}^{\left(n\right)}\left(t\right) & ={\left(\hat{T}\mathrm{exp}\left(-\frac{i}{\hbar }\int_{0}^{t}{\hat{H}}_{\mathrm{DQ},\mathrm{fl}}^{\left(n\right)}\left(t_{1}\right)dt_{1}\right)\right)}^{-1}\\ & {\hat{H}}_{\mathrm{DQ},yy}^{\left(n\right)}\left(t\right)\left(\hat{T}\mathrm{exp}\left(-\frac{i}{\hbar }\int_{0}^{t}{\hat{H}}_{\mathrm{DQ},\mathrm{fl}}^{\left(n\right)}\left(t_{1}\right)dt_{1}\right)\right). \end{array}$$

It is worth noting that the transition reflected in Eqs. (21) and (22) is analogous to the transition in Eqs. (13) and (14) and represents the second step in transitioning to the interaction representation that we are ultimately interested in. Substituting the relation Eq. (22) into (20), taking into account the identity (Eq. 7), we obtain

23 $$\begin{array}{rl} A_{n}\left(2\tau_{\mathrm{DQ}}\right) & =\frac{\beta \hbar \omega_{0}}{{\left(2I+1\right)}^{N_{s}}}\\ & \;\;{\langle {\mathrm{Tr}}_{s}\left({\hat{I}}^{z}{\hat{U}}_{2}^{\left(\mathrm{DQ},n\right)}\left(2\tau_{\mathrm{DQ}}\right){\hat{I}}^{z}{\hat{U}}_{2}^{*(\mathrm{DQ},n)}\left(2\tau_{\mathrm{DQ}}\right)\right)\rangle }_{\mathrm{eq}}, \end{array}$$

where

24 $${\hat{U}}_{2}^{\left(\mathrm{DQ},n\right)}\left(t\right)=\hat{T}\mathrm{exp}\left(-\frac{i}{\hbar }\int_{0}^{t}{\hat{\tilde{H}}}_{\mathrm{DQ},yy}^{\left(n\right)}\left(t_{1}\right)dt_{1}\right).$$

Note that in deriving relations (22) and (23), only a single asymptotically exact approximation, Eq. (15), is made.

### 2.2 The simplest approximation 

If we neglect the influence of flip-flop processes, i.e., putting ${\hat{H}}_{\mathrm{DQ},\mathrm{fl}}^{\left(n\right)}\left(t\right)=0$ in Eqs. (21) and (22), then with respect to the spin variables, Eq. (23) can be counted exactly if the dynamics of the lattice variables can be treated classically. Indeed, in this case, $\left[{\hat{H}}_{\mathrm{DQ},yy}^{\left(n\right)}\left(t_{2}\right);{\hat{H}}_{\mathrm{DQ},yy}^{\left(n\right)}\left(t_{1}\right)\right]=0$ and the propagator ${\hat{U}}_{2}^{\left(\mathrm{DQ},n\right)}\left(t\right)$ with respect to spin variables take a relatively simple form:

25 $$\begin{array}{rl} {\hat{U}}_{2}^{\left(\mathrm{DQ},n\right)}\left(t\right) & ≃\mathrm{exp}\left(-\frac{i}{\hbar }\int_{0}^{t}{\hat{\tilde{H}}}_{\mathrm{DQ},yy}^{\left(n\right)}\left(t_{1}\right)dt_{1}\right)\\ & =\mathrm{exp}\left(-i\sum_{i<j}2\varphi_{ij}^{\left(n\right)}\left(t\right){\hat{I}}_{i}^{y}{\hat{I}}_{j}^{y}\right), \end{array}$$

where

26 $$\varphi_{ij}^{\left(n\right)}\left(t\right)=\int_{0}^{t}\omega_{ij}^{\left(n\right)}\left(t_{1}\right)dt_{1}.$$

Substituting relations (25) and (26) into formula Eq. (20) using the algebraic properties of spin operators for I=1/2 (see details in Fatkullin et al., 2012, and Fatkullin et al., 2013), we obtain the following simplest approximation for experimentally observed DQ signals:

27 $$\begin{array}{rl} A_{n}^{\mathrm{sim}}\left(2\tau_{\mathrm{DQ}}\right) & =\frac{\beta \hbar \omega_{0}}{4}\sum_{k}{\langle \prod_{i}\mathrm{cos}\left(\varphi_{ik}^{\mathrm{ex}}+(-1{)}^{n}\varphi_{ik}^{\mathrm{rec}}\right)\rangle }_{\mathrm{eq}},\\ I_{\mathrm{nDQ}}^{\mathrm{sim}}\left(\tau_{\mathrm{DQ}}\right) & \equiv \frac{1}{2}\left(\frac{A_{1}\left(2\tau_{\mathrm{DQ}}\right)-A_{0}\left(2\tau_{\mathrm{DQ}}\right)}{A_{1}\left(2\tau_{\mathrm{DQ}}\right)}\right)\\ & =\frac{1}{2}\left(1-\frac{\sum_{i}{\langle \prod_{j}\mathrm{cos}\left(\varphi_{ij}^{\mathrm{ex}}+\varphi_{ij}^{\mathrm{rec}}\right)\rangle }_{\mathrm{eq}}}{\sum_{i}{\langle \prod_{j}\mathrm{cos}\left(\varphi_{ij}^{\mathrm{ex}}-\varphi_{ij}^{\mathrm{rec}}\right)\rangle }_{\mathrm{eq}}}\right), \end{array}$$

with

28 $$\varphi_{ik}^{\mathrm{ex}}=\int_{0}^{\tau_{\mathrm{DQ}}}\omega_{ik}\left(t_{1}\right)dt_{1},\varphi_{ik}^{\mathrm{rec}}=\int_{\tau_{\mathrm{DQ}}}^{2\tau_{\mathrm{DQ}}}\omega_{ik}\left(t_{1}\right)dt_{1}.$$

It is important to note that the derivation of relation (27) proposed in this paper, in contrast to the previous work (Fatkullin et al., 2013), does not use the Anderson–Weiss approximation directly. For a system of spin pairs, when i,k=1,2 , our Eq. (27) exactly recovers the known result:

29 $$A_{n}^{\mathrm{pair}}\left(2\tau_{\mathrm{DQ}}\right)=\frac{\beta \hbar \omega_{0}}{2}{\langle \mathrm{cos}\left(\varphi_{12}^{\mathrm{ex}}+{\left(-1\right)}^{n}\varphi_{12}^{\mathrm{rec}}\right)\rangle }_{\mathrm{eq}}.$$

### 2.3 Effect of flip-flop transitions

As already noted, flip-flop transitions are induced by the partial Hamiltonian ${\hat{H}}_{\mathrm{DQ},\mathrm{fl}}^{\left(n\right)}\left(t\right)$ given by Eq. (18). Their influence on the experimentally observed signal $A_{n}\left(2\tau_{\mathrm{DQ}}\right)$ is through the Hamiltonian ${\hat{\tilde{H}}}_{\mathrm{DQ},yy}^{\left(n\right)}\left(t\right)$ ; see relations (20)–(22), which can be rewritten in the following way:

30 $${\hat{\tilde{H}}}_{\mathrm{DQ},yy}^{\left(n\right)}\left(t\right)=\sum_{i<j}2\hbar \omega_{ij}^{\left(n\right)}\left(t\right){\left({\hat{I}}_{i}^{y}{\hat{I}}_{j}^{y}\right)}_{t}^{\mathrm{fl}},$$

where

31 $$\begin{array}{rl} {\left({\hat{I}}_{i}^{y}{\hat{I}}_{j}^{y}\right)}_{t}^{\mathrm{fl}}\equiv & {\left(\hat{T}\mathrm{exp}\left(-\frac{i}{\hbar }\int_{0}^{t}{\hat{H}}_{\mathrm{DQ},\mathrm{fl}}^{\left(n\right)}\left(t_{1}\right)dt_{1}\right)\right)}^{-1}\\ & {\hat{I}}_{i}^{y}{\hat{I}}_{j}^{y}\left(\hat{T}\mathrm{exp}\left(-\frac{i}{\hbar }\int_{0}^{t}{\hat{H}}_{\mathrm{DQ},\mathrm{fl}}^{\left(n\right)}\left(t_{1}\right)dt_{1}\right)\right). \end{array}$$

The approximation considered earlier, in Sect. 2.2, is equivalent to neglecting the dependence of the operator ${\left({\hat{I}}_{i}^{y}{\hat{I}}_{j}^{y}\right)}_{t}^{\mathrm{fl}}$ on time. In this section, we consider the approximation by its projection in Liouville spin space to the initial value:

32 $$\begin{array}{rl} {\left\{{\hat{I}}_{i}^{y}{\hat{I}}_{j}^{y}\right\}}_{t}^{\mathrm{fl},n} & ≃{\hat{I}}_{i}^{y}{\hat{I}}_{j}^{y}\frac{{\mathrm{Tr}}_{s}\left({\hat{I}}_{i}^{y}{\hat{I}}_{j}^{y}{\left\{{\hat{I}}_{i}^{y}{\hat{I}}_{j}^{y}\right\}}_{t}^{\mathrm{fl}}\right)}{{\mathrm{Tr}}_{s}\left({\left({\hat{I}}_{i}^{y}\right)}^{2}{\left({\hat{I}}_{j}^{y}\right)}^{2}\right)}\\ & \equiv {\tilde{P}}_{ij}^{n,\mathrm{fl}}\left\{t;0\right\}{\hat{I}}_{i}^{y}{\hat{I}}_{j}^{y}. \end{array}$$

The quantity ${\tilde{P}}_{ij}^{n,\mathrm{fl}}\left\{t;0\right\}$ does not depend on spin variables, but it is a complex function of time-dependent lattice variables. Later we see that after averaging over the lattice variables, it can be, for the case of n=0 , viewed as the probability that during the time interval t , none of the spins in question with numbers i and j participated in the flip-flop process with other spins.

Consider the expansion of the value ${\tilde{P}}_{ij}^{n,\mathrm{fl}}\left\{t_{2};t_{1}\right\}$ in a perturbation theory series with respect to ${\hat{H}}_{\mathrm{DQ},\mathrm{fl}}^{\left(n\right)}\left(t_{1}\right)$ :

33 $$\begin{array}{rl} {\tilde{P}}_{ij}^{n,\mathrm{fl}}\left\{t_{2};t_{1}\right\} & =1-\frac{i}{2}\int_{t_{1}}^{t_{2}}d\tau_{1}\sum_{k,l}\omega_{kl}^{\left(n\right)}\left(\tau_{1}\right)\\ & \;\;\frac{{\mathrm{Tr}}_{s}\left({\hat{I}}_{i}^{y}{\hat{I}}_{j}^{y}\left[{\hat{I}}_{k}^{x}{\hat{I}}_{l}^{x}+{\hat{I}}_{k}^{y}{\hat{I}}_{l}^{y};{\hat{I}}_{i}^{y}{\hat{I}}_{j}^{y}\right]\right)}{{\mathrm{Tr}}_{s}\left({\left({\hat{I}}_{i}^{y}\right)}^{2}{\left({\hat{I}}_{j}^{y}\right)}^{2}\right)}\\ & -\frac{1}{4}\int_{t_{1}}^{t_{2}}d\tau_{2}\int_{t_{1}}^{\tau_{2}}d\tau_{1}\sum_{k,l;s,t}\omega_{kl}^{\left(n\right)}\left(\tau_{2}\right)\omega_{\mathrm{st}}^{\left(n\right)}\left(\tau_{1}\right)\\ & \;\;\frac{{\mathrm{Tr}}_{s}\left({\hat{I}}_{i}^{y}{\hat{I}}_{j}^{y}\left[{\hat{I}}_{k}^{x}{\hat{I}}_{l}^{x}+{\hat{I}}_{k}^{y}{\hat{I}}_{l}^{y};\left[{\hat{I}}_{s}^{x}{\hat{I}}_{t}^{x}+{\hat{I}}_{s}^{y}{\hat{I}}_{t}^{y};{\hat{I}}_{i}^{y}{\hat{I}}_{j}^{y}\right]\right]\right)}{{\mathrm{Tr}}_{s}\left({\left({\hat{I}}_{i}^{y}\right)}^{2}{\left({\hat{I}}_{j}^{y}\right)}^{2}\right)}\\ & +\ldots \end{array}$$

The first-order contribution by $\omega_{kl}^{\left(n\right)}\left(\tau_{1}\right)$ in relation (33) turns out to be exactly 0:

34 $$\begin{array}{rl} & {\mathrm{Tr}}_{s}\left({\hat{I}}_{i}^{y}{\hat{I}}_{j}^{y}\left[{\hat{I}}_{k}^{x}{\hat{I}}_{l}^{x}+{\hat{I}}_{k}^{y}{\hat{I}}_{l}^{y};{\hat{I}}_{i}^{y}{\hat{I}}_{j}^{y}\right]\right)\\ & \;\;\;=-{\mathrm{Tr}}_{s}\left({\hat{I}}_{i}^{y}{\hat{I}}_{j}^{y}\left[{\hat{I}}_{i}^{y}{\hat{I}}_{j}^{y};{\hat{I}}_{k}^{x}{\hat{I}}_{l}^{x}+{\hat{I}}_{k}^{y}{\hat{I}}_{l}^{y}\right]\right)\\ & \;\;\;={\mathrm{Tr}}_{s}\left(\left[{\hat{I}}_{i}^{y}{\hat{I}}_{j}^{y};{\hat{I}}_{i}^{y}{\hat{I}}_{j}^{y}\right];{\hat{I}}_{k}^{x}{\hat{I}}_{l}^{x}+{\hat{I}}_{k}^{y}{\hat{I}}_{l}^{y}\right)=0. \end{array}$$

The relation is thus simplified:

35 $$\begin{array}{rl} {\tilde{P}}_{ij}^{\left(n\right)\mathrm{fl}}\left(t_{2};t_{1}\right) & =1-\frac{1}{4}\int_{t_{1}}^{t_{2}}d\tau_{2}\int_{t_{1}}^{\tau_{2}}d\tau_{1}\sum_{k,l;s,t}\omega_{kl}^{\left(n\right)}\left(\tau_{2}\right)\omega_{\mathrm{st}}^{\left(n\right)}\left(\tau_{1}\right)\\ & \;\;\;\;\frac{{\mathrm{Tr}}_{s}\left(\left[{\hat{I}}_{i}^{y}{\hat{I}}_{j}^{y};{\hat{I}}_{k}^{x}{\hat{I}}_{l}^{x}\right]\left[{\hat{I}}_{s}^{x}{\hat{I}}_{t}^{x};{\hat{I}}_{i}^{y}{\hat{I}}_{j}^{y}\right]\right)}{{\mathrm{Tr}}_{s}\left({\left({\hat{I}}_{i}^{y}\right)}^{2}{\left({\hat{I}}_{j}^{y}\right)}^{2}\right)}\\ & +\ldots \end{array}$$

The standard commutator and trace calculations on the right-hand side lead to the following result:

36 $$\begin{array}{rl} & \sum_{k,l;s,t}\omega_{kl}^{\left(n\right)}\left(\tau_{2}\right)\omega_{\mathrm{st}}^{\left(n\right)}\left(\tau_{1}\right)\frac{{\mathrm{Tr}}_{s}\left(\left[{\hat{I}}_{i}^{y}{\hat{I}}_{j}^{y};{\hat{I}}_{k}^{x}{\hat{I}}_{l}^{x}\right]\left[{\hat{I}}_{s}^{x}{\hat{I}}_{t}^{x};{\hat{I}}_{i}^{y}{\hat{I}}_{j}^{y}\right]\right)}{{\mathrm{Tr}}_{s}\left({\left({\hat{I}}_{i}^{y}\right)}^{2}{\left({\hat{I}}_{j}^{y}\right)}^{2}\right)}\\ & \;\;\;=\frac{4}{3}I\left(I+1\right)\sum_{k}{}^{\prime }\left(\omega_{ik}^{\left(n\right)}\left(\tau_{2}\right)\omega_{ik}^{\left(n\right)}\left(\tau_{1}\right)+\omega_{jk}^{\left(n\right)}\left(\tau_{2}\right)\omega_{jk}^{\left(n\right)}\left(\tau_{1}\right)\right)\\ & \;\;\;+\frac{8}{5}\left\{I\left(I+1\right)-\frac{3}{4}\right\}\omega_{ij}^{\left(n\right)}\left(\tau_{2}\right)\omega_{ij}^{\left(n\right)}\left(\tau_{1}\right), \end{array}$$

where $\sum_{k}{}^{\prime }\ldots$ implies that summation is performed with the restrictions k≠i,j .

For the case of spins I = 1/2 , the last term on the right-hand side of relation (34) is exactly 0. This is expected because the mutual flip-flop transitions between spins with numbers i and j do not change the value of the product ${\hat{I}}_{i}^{y}{\hat{I}}_{j}^{y}$ in the considered case. At the same time, flip-flop transitions with other system spins are accounted for in the terms proportional to $\omega_{ik}^{\left(n\right)}\left(\tau_{2}\right)\omega_{ik}^{\left(n\right)}\left(\tau_{1}\right)+\omega_{jk}^{\left(n\right)}\left(\tau_{2}\right)\omega_{jk}^{\left(n\right)}\left(\tau_{1}\right)$ , which change the value of ${\hat{I}}_{i}^{y}{\hat{I}}_{j}^{y}$ .

Hereafter, we assume that the motion of the lattice variables is correctly described by classical dynamics, which allows us to neglect the explicit time ordering of the corresponding variables; relation (35) takes the following form:

37 $$\begin{array}{rl} {\tilde{P}}_{ij}^{n,\mathrm{fl}}\left\{t_{2};t_{1}\right\} & =1-\frac{I\left(I+1\right)}{3}\int_{t_{1}}^{t_{2}}d\tau_{2}\int_{t_{1}}^{\tau_{2}}d\tau_{1}\sum_{k}{}^{\prime }\\ & \;\;\;\;\left(\omega_{ik}^{\left(n\right)}\left(\tau_{2}\right)\omega_{ik}^{\left(n\right)}\left(\tau_{1}\right)+\omega_{jk}^{\left(n\right)}\left(\tau_{2}\right)\omega_{jk}^{\left(n\right)}\left(\tau_{1}\right)\right)\\ & +\ldots \\ & =1-\frac{1}{6}I\left(I+1\right)\sum_{k}{}^{\prime }\\ & \;\;\;\;\left({\left(\varphi_{ik}^{\left(n\right)}\left(t_{2};t_{1}\right)\right)}^{2}+{\left(\varphi_{jk}^{\left(n\right)}\left(t_{2};t_{1}\right)\right)}^{2}\right)\\ & +\ldots , \end{array}$$

where $\varphi_{\mathrm{st}}^{\left(n\right)}\left(t_{2};t_{1}\right)=\int_{t_{1}}^{t_{2}}\omega_{\mathrm{st}}^{\left(n\right)}\left(\tau \right)d\tau$ .

Note that in the right-hand side of Eq. (32) we have taken into account only projections of the operator ${\left({\hat{I}}_{i}^{y}{\hat{I}}_{j}^{y}\right)}_{t}^{\mathrm{fl}}$ on the initial spin operator ${\hat{I}}_{i}^{y}{\hat{I}}_{j}^{y}$ and neglected its projections on the spin operators such as ${\hat{I}}_{k}^{y}{\hat{I}}_{l}^{y}$ for cases when k≠i,j or l≠i,j . By direct and lengthy calculations analogous to Eqs. (33)–(34), one can see that in the second order of perturbation theory in $\omega_{kl}^{\left(n\right)}\left(\tau \right)$ , their contributions to Eq. (35) are exactly zero.

In further calculations, we will apply the Anderson–Weiss approximation with respect to the magnitude ${\tilde{P}}_{ij}^{n,\mathrm{fl}}\left\{t_{2};t_{1}\right\}$ :

38 $$\begin{array}{rl} {\tilde{P}}_{ij}^{n,\mathrm{fl}}\left\{t_{2};t_{1}\right\} & =\mathrm{exp}\{-\frac{1}{6}I\left(I+1\right)\sum_{k}{}^{\prime }({\left(\varphi_{ik}^{\left(n\right)}\left(t_{2};t_{1}\right)\right)}^{2}\\ & +{\left(\varphi_{jk}^{\left(n\right)}\left(t_{2};t_{1}\right)\right)}^{2})\}. \end{array}$$

It seems appropriate to note that the approximation equation, Eq. (32), reconstructs the formal mathematical structure of the previously studied Hamiltonian Eq. (16) by modifying in it only the time-dependent spin–lattice interaction constant:

39 $${\hat{\tilde{H}}}_{\mathrm{DQ},yy}^{\left(n\right)}\left(t\right)≃\sum_{i<j}2\hbar {\tilde{\omega }}_{ij}^{\left(n\right)}\left(t\right){\hat{I}}_{i}^{y}{\hat{I}}_{j}^{y},$$

with

40 $${\tilde{\omega }}_{ij}^{\left(n\right)}\left(t\right)={\tilde{P}}_{ij}^{n,\mathrm{fl}}\left(t;0\right)\omega_{ij}^{\left(n\right)}\left(t\right).$$

Effects associated with the Hamiltonian Eq. (18) are now considered by the presence of a multiplier ${\tilde{P}}_{ij}^{\mathrm{fl}}\left(t;0\right)$ . Therefore, by analogy with relation (27), we can immediately write down the relations for experimentally observed signals:

41 $$A_{n}\left(2\tau_{\mathrm{DQ}}\right)=\frac{\beta \hbar \omega_{0}}{4}\sum_{k}{\langle \prod_{i}\mathrm{cos}\left({\tilde{\varphi }}_{ik}^{\mathrm{ex}}+{\left(-1\right)}^{n}{\tilde{\varphi }}_{ik}^{\mathrm{rec}}\right)\rangle }_{\mathrm{eq}},$$

with

42 $${\tilde{\varphi }}_{ik}^{\mathrm{ex}}=\int_{0}^{\tau_{\mathrm{DQ}}}{\tilde{\omega }}_{ik}(t_{1})\;dt_{1},{\tilde{\varphi }}_{ik}^{\mathrm{rec}}=\int_{\tau_{\mathrm{DQ}}}^{2\tau_{\mathrm{DQ}}}{\tilde{\omega }}_{ik}(t_{1})\;dt_{1}.$$

From the experimentally measured quantities $A_{0}\left(2\tau_{\mathrm{DQ}}\right)$ and $A_{1}\left(2\tau_{\mathrm{DQ}}\right)$ , one can construct the so-called normalized DQ (nDQ) buildup function:

43 $$I_{\mathrm{nDQ}}\left(\tau_{\mathrm{DQ}}\right)=\frac{1}{2}\frac{A_{1}\left(2\tau_{\mathrm{DQ}}\right)-A_{0}\left(2\tau_{\mathrm{DQ}}\right)}{A_{1}\left(2\tau_{\mathrm{DQ}}\right)}.$$

Using Eq. (41), we get

44 $$I_{\mathrm{nDQ}}\left(\tau_{\mathrm{DQ}}\right)=\frac{1}{2}\left(1-\frac{\sum_{i}{\langle \prod_{j}\mathrm{cos}\left({\tilde{\varphi }}_{ij}^{\left(0\right),\mathrm{ex}}+{\tilde{\varphi }}_{ij}^{\left(0\right),rec}\right)\rangle }_{\mathrm{eq}}}{\sum_{i}{\langle \prod_{j}\mathrm{cos}\left({\tilde{\varphi }}_{ij}^{\left(1\right),\mathrm{ex}}-{\tilde{\varphi }}_{ij}^{\left(1\right),rec}\right)\rangle }_{\mathrm{eq}}}\right).$$

### 2.4 Interpretation of ${\tilde{P}}_{ij}^{n,\mathrm{fl}}\{t_{2};t_{1}\}$

The quantity under consideration is defined by relation (32); Eq. (37) is its Taylor expansion in ${\hat{H}}_{\mathrm{DQ},\mathrm{fl}}^{\left(n\right)}\left(t\right)$ , and Eq. (38) is in turn similar to its Anderson–Weiss approximation. Averaging in all the indicated relations was carried out only over spin variables. Therefore, according to Eq. (31), ${\tilde{P}}_{ij}^{n,\mathrm{fl}}\left\{t_{2};t_{1}\right\}$ remains a function of lattice variables, i.e., it depends, in general, on the spatial coordinates of spins at all previous time moments. It is not in itself experimentally measurable. Experimentally measured quantities that depend on ${\tilde{P}}_{ij}^{n,\mathrm{fl}}\left\{t_{2};t_{1}\right\}$ , as follows from Eqs. (19), (43), (44), contain an additional averaging over the equilibrium distribution of lattice variables. In mathematical terms, the quantity ${\tilde{P}}_{ij}^{n,\mathrm{fl}}\left\{t_{2};t_{1}\right\}$ is a multi-dimensional random process, microscopically defined by the Hamiltonian of lattice variables ${\hat{H}}_{L}$ .

After averaging Eq. (37) over the lattice variables, we obtain

45 $$\begin{array}{rl} P_{ij}^{n,\mathrm{fl}}\left\{t_{2};t_{1}\right\} & \equiv {\langle {\tilde{P}}_{ij}^{n,\mathrm{fl}}\left\{t_{2};t_{1}\right\}\rangle }_{\mathrm{eq}}\\ & =1-\frac{I\left(I+1\right)}{3}\int_{t_{1}}^{t_{2}}d\tau_{2}\int_{t_{1}}^{\tau_{2}}d\tau_{1}\sum_{k}{}^{\prime }\\ & \;\;\;\;\left({\langle \omega_{ik}^{\left(n\right)}\left(\tau_{2}\right)\omega_{ik}^{\left(n\right)}\left(\tau_{1}\right)+\omega_{jk}^{\left(n\right)}\left(\tau_{2}\right)\omega_{jk}^{\left(n\right)}\left(\tau_{1}\right)\rangle }_{\mathrm{eq}}\right)\\ & +\ldots \\ & =1-\frac{1}{6}I\left(I+1\right)\sum_{k}{}^{\prime }({\langle {\left(\varphi_{ik}^{\left(n\right)}\left(t_{2};t_{1}\right)\right)}^{2}\rangle }_{\mathrm{eq}}\\ & +{\langle {\left(\varphi_{jk}^{\left(n\right)}\left(t_{2};t_{1}\right)\right)}^{2}\rangle }_{\mathrm{eq}})\\ & +\ldots \end{array}$$

Using the Anderson–Weiss approximation for this relation, we obtain

46 $$\begin{array}{rl} P_{ij}^{n,\mathrm{fl}}\left\{t_{2};t_{1}\right\} & =\mathrm{exp}\{-\frac{1}{6}I\left(I+1\right)\sum_{k}{}^{\prime }({\langle {\left(\varphi_{ik}^{\left(n\right)}\left(t_{2};t_{1}\right)\right)}^{2}\rangle }_{\mathrm{eq}}\\ & +{\langle {\left(\varphi_{jk}^{\left(n\right)}\left(t_{2};t_{1}\right)\right)}^{2}\rangle }_{\mathrm{eq}})\}. \end{array}$$

An expression similar to Eqs. (45) and (46) with numerical multiplier accuracy was obtained in Fatkullin et al. (2012), which discussed the modified Anderson–Weiss approximation with respect to the free induction decay (FID) signal of the proton spin system:

47 $$\begin{array}{rl} P_{kl}^{\mathrm{FID},\mathrm{fl}}\left(t\right) & =\mathrm{exp}\{-\int_{0}^{t}d\tau \left(t-\tau \right)\frac{I\left(I+1\right)}{6\hbar^{2}}\sum_{m}{}^{\prime }({\langle {\tilde{A}}_{km}\left(t\right){\tilde{A}}_{km}\left(0\right)\rangle }_{\mathrm{eq}}\\ & +{\langle {\tilde{A}}_{lm}\left(t\right){\tilde{A}}_{lm}\left(0\right)\rangle }_{\mathrm{eq}})\}, \end{array}$$

where ${\tilde{A}}_{kl}\left(t\right)=A_{kl}\left(t\right)-2J_{kl}\left(t\right)$ . $J_{kl}$ is the J coupling constant between spins with numbers k and l (here correcting a misprint of the numerical coefficient before the coupling constant in Fatkullin et al., 2012) and $A_{kl}\left(t\right)=\frac{\gamma^{2}\hbar^{2}}{r_{kl}^{3}\left(t\right)}\left(1-3{\mathrm{cos}}^{2}\left(\theta_{kl}\left(t\right)\right)\right)$ with variables identical to Eq. (2) in this paper. The value $P_{kl}^{\mathrm{FID},\mathrm{fl}}\left(t\right)$ can be viewed as the probability that a given pair of numbered k and l spins will not participate in flip-flop processes over time t with any third spin of the system.

In terms of the present paper, $J_{kl}=0$ and $A_{kl}\left(t\right)=2\omega_{kl}\left(t\right)$ . To consider motions of the lattice variables as classical, Eq. (47) can be rewritten in the following way:

48 $$\begin{array}{rl} P_{kl}^{\mathrm{FID},\mathrm{fl}}\left(t\right) & =\mathrm{exp}\{-\int_{0}^{t}d\tau \left(t-\tau \right)\frac{2I\left(I+1\right)}{3\hbar^{2}}\sum_{m}{}^{\prime }\\ & \;\;\;\;({\langle \omega_{km}\left(\tau \right)\omega_{km}\left(0\right)\rangle }_{\mathrm{eq}}+{\langle \omega_{lm}\left(\tau \right)\omega_{lm}\left(0\right)\rangle }_{\mathrm{eq}})\}\\ & =\mathrm{exp}\{-\frac{1}{3}I\left(I+1\right)\sum_{m}{}^{\prime }({\langle {\left(\varphi_{km}^{\left(0\right)}\left(t;0\right)\right)}^{2}\rangle }_{\mathrm{eq}}\\ & +{\langle {\left(\varphi_{lm}^{\left(0\right)}\left(t;0\right)\right)}^{2}\rangle }_{\mathrm{eq}})\}. \end{array}$$

The difference in the numerical coefficients 1/6 and 1/3 of the ratios in Eqs. (46) and (48), respectively, are noticeable. It is related to the fact that in the first case, the flip-flop processes are induced by the modified RF irradiated Hamiltonian Eq. (4), and in the second case by the secular part of the Hamiltonian of magnetic dipole–dipole interactions (Eq. 1). The indicated difference in the numerical coefficients indicates that in the case of DQ experiments, the influence of flip-flop processes is weaker than for FID or Hahn echo and will appear at later times. To complete the picture, it seems appropriate to quote an expression from Fatkullin et al. (2012) for the probability that spin number k will not participate in flip-flop processes at intervals t :

49 $$\begin{array}{rl} P_{k}^{\mathrm{FID},\mathrm{fl}}\left(t\right) & =\mathrm{exp}\{-\int_{0}^{t}d\tau \left(t-\tau \right)\frac{I\left(I+1\right)}{6\hbar^{2}}\sum_{m}{}^{\prime }\\ & \;\;\;\;{\langle {\tilde{A}}_{km}\left(\tau \right){\tilde{A}}_{km}\left(0\right)\rangle }_{\mathrm{eq}}\}\\ & =\mathrm{exp}\left\{-\frac{I\left(I+1\right)}{3}\sum_{m}{}^{\prime }{\langle {\left(\varphi_{km}^{\mathrm{ex}}\left(t;0\right)\right)}^{2}\rangle }_{\mathrm{eq}}\right\}, \end{array}$$

where the second line on the right-hand side is rewritten in terms of the variables of this paper.

From these examples, it seems to us that the value ${\tilde{P}}_{ij}^{n,\mathrm{fl}}\left\{t_{2};t_{1}\right\}$ for the case when n=0 can be regarded as a conditional probability for spins with numbers i and j not to participate in flip-flop processes with other system spins during the time interval $t_{1}\leq t\leq t_{2}$ , provided that the lattice variables change along the phase trajectory defined by a particular set of lattice coordinates during the specified time interval.

### 2.5 The case of a (quasi-)rigid lattice and Anderson–Weiss approximation

In this case, spins undergo only small oscillations in the vicinity of the equilibrium positions. Alternatively, the case also applies to anisotropic fast-limit motions (such as in polymer networks at high temperatures), where the dipolar couplings are replaced by quasi-static and possibly rather small residual dipolar couplings of order $3\omega_{ij}/\left(5N\right)$ , where N is the number of statistical segments between cross-links (Kuhn and Grün, 1942). Also in this case, relevant oscillations around the mean value of $3\omega_{ij}/\left(5N\right)$ are rather small for relevant long timescales. In both cases, the time dependence of frequencies $\omega_{ij}\left(t\right)$ in the Hamiltonian Eq. (4) can be neglected, and consequently $\omega_{ij}^{\left(n\right)}\left(t\right)={\left(-1\right)}^{n\theta \left(t-\tau_{\mathrm{DQ}}\right)}\omega_{ij}$ . Then, the signal $A_{1}\left(t\right)$ is always fully recovered at time $t=2\tau_{\mathrm{DQ}}$ . Our approximation, Eq. (32), preserves this property. This follows from relations (37) and (38) since, as is easy to see, we have for $0\leq t\leq \tau_{\mathrm{DQ}}$

50 $${\tilde{P}}_{ij}^{1,\mathrm{fl}}\left\{t+\tau_{\mathrm{DQ}};0\right\}={\tilde{P}}_{ij}^{1,\mathrm{fl}}\left\{\tau_{\mathrm{DQ}}-t;0\right\}$$

and therefore

51 $$\begin{array}{rl} {\tilde{\varphi }}_{ij}^{\left(1\right),\mathrm{ex}}-{\tilde{\varphi }}_{ij}^{\left(1\right),\mathrm{rec}} & =\omega_{ij}\int_{0}^{\tau_{\mathrm{DQ}}}({\tilde{P}}_{ij}^{1,\mathrm{fl}}\left\{t_{1}+\tau_{\mathrm{DQ}};0\right\}\\ & -{\tilde{P}}_{ij}^{1,\mathrm{fl}}\left\{\tau_{\mathrm{DQ}}-t_{1};0\right\})dt_{1}=0. \end{array}$$

Note that this property is not trivial at all since the flip-flop processes are elementary steps of spin diffusion, which is irreversible for the signal $A_{0}\left(2\tau_{\mathrm{DQ}}\right)$ and reversible in time for the signal $A_{1}\left(2\tau_{\mathrm{DQ}}\right)$ . This property allows us to rewrite relation (44) for the rigid lattice as follows:

52 $$I_{\mathrm{nDQ}}^{\mathrm{rig}}\left(\tau_{\mathrm{DQ}}\right)=\frac{1}{2}\left(1-\frac{1}{N_{s}}\sum_{i}{\langle \prod_{j}\mathrm{cos}\left({\tilde{\varphi }}_{ij}^{\mathrm{ex}}+{\tilde{\varphi }}_{ij}^{\mathrm{rec}}\right)\rangle }_{\mathrm{eq}}\right).$$

Using Eq. (42), we can rewrite Eq. (52) as follows:

53 $$I_{\mathrm{nDQ}}^{\mathrm{rig}}\left(\tau_{\mathrm{DQ}}\right)=\frac{1}{2}\left(1-\frac{1}{N_{s}}\sum_{i}{\langle \prod_{j}\mathrm{cos}\left(\left(\int_{0}^{2\tau_{\mathrm{DQ}}}{\tilde{\omega }}_{ij}^{\left(0\right)}(t_{1})dt_{1}\right)\right)\rangle }_{\mathrm{eq}}\right).$$

Let us now consider the following quantity:

54 $$\prod_{i}\left(2\tau_{\mathrm{DQ}}\right)\equiv {\langle \prod_{j}\mathrm{cos}\left(\left(\int_{0}^{2\tau_{\mathrm{DQ}}}{\tilde{\omega }}_{ij}^{\left(0\right)}(t_{1})dt_{1}\right)\right)\rangle }_{\mathrm{eq}}.$$

Decomposing the right-hand side into a Taylor expansion, we obtain

55 $$\prod_{i}\left(2\tau_{\mathrm{DQ}}\right)=\prod_{j}\left(1-\frac{1}{2!}\int_{0}^{2\tau_{\mathrm{DQ}}}dt_{2}\int_{0}^{2\tau_{\mathrm{DQ}}}dt_{1}{\langle {\tilde{\omega }}_{ij}^{\left(0\right)}(t_{2}){\tilde{\omega }}_{ij}^{\left(0\right)}(t_{1})\rangle }_{\mathrm{eq}}+\ldots \right).$$

Considering the quantity ${\tilde{\omega }}_{ij}^{\left(0\right)}(t_{1})$ as stochastic stationary random processes whose correlation functions have symmetry with respect to time reversal, we obtain

56 $$\begin{array}{rl} \prod_{i}\left(2\tau_{\mathrm{DQ}}\right) & =\prod_{j}\left(1-\frac{1}{2!}\int_{0}^{2\tau_{\mathrm{DQ}}}dt_{2}\int_{0}^{2\tau_{\mathrm{DQ}}}dt_{1}{\langle {\tilde{\omega }}_{ij}^{\left(0\right)}(t_{2}-t_{1}){\tilde{\omega }}_{ij}^{\left(0\right)}(0)\rangle }_{\mathrm{eq}}+\ldots \right)\\ & =\prod_{j}\left(1-\int_{0}^{2\tau_{\mathrm{DQ}}}d\tau \left(2\tau_{\mathrm{DQ}}-\tau \right){\langle {\tilde{P}}_{ij}^{0,\mathrm{fl}}\left(\tau ;0\right){\tilde{P}}_{ij}^{0,\mathrm{fl}}\left(0;0\right)\omega_{ij}^{\left(0\right)}(\tau )\omega_{ij}^{\left(0\right)}(0)\rangle }_{\mathrm{eq}}+\ldots \right). \end{array}$$

Then we can apply a mean-field-like approximation as follows:

57 $$\begin{array}{rl} \prod_{i}\left(2\tau_{\mathrm{DQ}}\right)= & \prod_{j}(1-\int_{0}^{2\tau_{\mathrm{DQ}}}d\tau \left(2\tau_{\mathrm{DQ}}-\tau \right)\\ & \;\;\;{\langle {\tilde{P}}_{ij}^{0,\mathrm{fl}}\left(\tau ;0\right){\tilde{P}}_{ij}^{0,\mathrm{fl}}\left(0;0\right)\omega_{ij}^{\left(0\right)}(\tau )\omega_{ij}^{\left(0\right)}(0)\rangle }_{\mathrm{eq}}+\ldots \;)\\ & ≃\prod_{j}(1-\int_{0}^{2\tau_{\mathrm{DQ}}}d\tau \left(2\tau_{\mathrm{DQ}}-\tau \right)\\ & \;\;\;{\langle {\tilde{P}}_{ij}^{0,\mathrm{fl}}\left(\tau ;0\right){\tilde{P}}_{ij}^{0,\mathrm{fl}}\left(0;0\right)\rangle }_{\mathrm{eq}}{\langle \omega_{ij}^{\left(0\right)}(\tau )\omega_{ij}^{\left(0\right)}(0)\rangle }_{\mathrm{eq}}+\ldots ). \end{array}$$

By definition (see Eq. 32), we have ${\tilde{P}}_{ij}^{0,\mathrm{fl}}\left(0;0\right)=1$ . Therefore, we can rewrite Eq. (57) as follows:

58 $$\begin{array}{rl} \prod_{i}\left(2\tau_{\mathrm{DQ}}\right) & ≃\prod_{j}(1-\int_{0}^{2\tau_{\mathrm{DQ}}}d\tau \left(2\tau_{\mathrm{DQ}}-\tau \right)\\ & \;\;\;{\langle {\tilde{P}}_{ij}^{0,\mathrm{fl}}\left(\tau ;0\right)\rangle }_{\mathrm{eq}}{\langle \omega_{ij}^{\left(0\right)}(\tau )\omega_{ij}^{\left(0\right)}(0)\rangle }_{\mathrm{eq}}+\ldots )\\ & =\prod_{j}(1-\int_{0}^{2\tau_{\mathrm{DQ}}}d\tau \left(2\tau_{\mathrm{DQ}}-\tau \right)P_{ij}^{0,\mathrm{fl}}\left(\tau ;0\right)\\ & \;\;\;{\langle \omega_{ij}^{\left(0\right)}(\tau )\omega_{ij}^{\left(0\right)}(0)\rangle }_{\mathrm{eq}}+\ldots ). \end{array}$$

Remembering that we started with the approximation of the product of cosines (see relation 54) and remembering that for a rigid lattice $\omega_{ij}^{\left(0\right)}\left(t\right)=\omega_{ij}$ , it is natural to use the following approximation for the original relation (53):

59 $$\begin{array}{rl} & I_{\mathrm{nDQ}}^{\mathrm{rig}}\left(\tau_{\mathrm{DQ}}\right)=\frac{1}{2}(1-\frac{1}{N_{s}}\sum_{i}\prod_{j}\\ & \;\;\;\mathrm{cos}\left(\sqrt{2\omega_{ij}^{2}\int_{0}^{2\tau_{\mathrm{DQ}}}d\tau \left(2\tau_{\mathrm{DQ}}-\tau \right)P_{ij}^{0,\mathrm{fl}}\left(\tau ;0\right)}\right)). \end{array}$$

Using the approximation in Eq. (46) for the value $P_{ij}^{0,\mathrm{fl}}\left(\tau ;0\right)$ , after a number of steps, Eq. (59) is transformed into the following form:

60 $$I_{\mathrm{nDQ}}^{rig}\left(\tau_{\mathrm{DQ}}\right)=\frac{1}{2}\left(1-\frac{1}{N_{s}}\sum_{i}\prod_{j}\mathrm{cos}\left(\omega_{ij}T_{ij}\right)\right),$$

with

61 $$\begin{array}{rl} \Omega_{ij}^{2} & \equiv \frac{I\left(I+1\right)}{6}\left(\sum_{k}{}^{\prime }\left(\omega_{ik}^{2}+\omega_{jk}^{2}\right)\right),\\ T_{ij} & =\sqrt{\frac{2\sqrt{\pi }\tau_{\mathrm{DQ}}}{\Omega_{ij}}\mathrm{erf}\left(2\Omega_{ij}\tau_{\mathrm{DQ}}\right)-\frac{1}{\Omega_{ij}^{2}}\left(1-\mathrm{exp}\left(-4\Omega_{ij}^{2}\tau_{\mathrm{DQ}}^{2}\right)\right)}. \end{array}$$

Equation (60) has the following asymptotic values:

62 $$I_{\mathrm{nDQ}}^{rig}\left(\tau_{\mathrm{DQ}}\right)=\left\{\begin{array}{l} \frac{1}{N_{s}}\sum_{i,j}\omega_{ij}^{2}\tau_{\mathrm{DQ}}^{2}\;\;\;\text{for}\;\;\;\tau_{\mathrm{DQ}}\ll \Omega_{ij}^{-1}\propto T_{2}^{\mathrm{eff}},\\ \frac{1}{2}\left(1-\frac{1}{N_{s}}\sum_{i}\prod_{j}\mathrm{cos}\left(\omega_{ij}\sqrt{\frac{2\sqrt{\pi }\tau_{\mathrm{DQ}}}{\Omega_{ij}}}\right)\right)\;\;\;\text{for}\;\;\;\tau_{\mathrm{DQ}}\gg \Omega_{ij}^{-1}\propto T_{2}^{\mathrm{eff}}. \end{array}\right.$$

For cases in which the spatial displacements of spins during DQ experiments cannot be neglected, the Anderson–Weiss approximation is usually used. With respect to our Eq. (44), it leads to the following formula:

63 $$I_{\mathrm{nDQ}}\left(\tau_{\mathrm{DQ}}\right)=\frac{1}{2}\left(1-\frac{\sum_{i}\mathrm{exp}\left\{-\frac{1}{2}\sum_{j}{\langle {\left({\tilde{\varphi }}_{ij}^{\left(0\right),\mathrm{ex}}+{\tilde{\varphi }}_{ij}^{\left(0\right),rec}\right)}^{2}\rangle }_{\mathrm{eq}}\right\}}{\sum_{i}\mathrm{exp}\left\{-\frac{1}{2}\sum_{j}{\langle {\left({\tilde{\varphi }}_{ij}^{\left(1\right),\mathrm{ex}}-{\tilde{\varphi }}_{ij}^{\left(1\right),rec}\right)}^{2}\rangle }_{\mathrm{eq}}\right\}}\right).$$

If all spins have an equivalent environment, then the expression is simplified:

64 $$\begin{array}{rl} I_{\mathrm{nDQ}}\left(\tau_{\mathrm{DQ}}\right) & =\frac{1}{2}(1-\mathrm{exp}\{-\sum_{j}({\langle {\tilde{\varphi }}_{ij}^{\left(0\right),\mathrm{ex}}{\tilde{\varphi }}_{ij}^{\left(0\right),rec}\rangle }_{\mathrm{eq}}\\ & +{\langle {\tilde{\varphi }}_{ij}^{\left(1\right),\mathrm{ex}}{\tilde{\varphi }}_{ij}^{\left(1\right),rec}\rangle }_{\mathrm{eq}})\}). \end{array}$$

### 2.6 Estimation of characteristic flip-flop transition times in DQ experiments

As noted at the beginning of Sect. 2.4, the new expressions for the DQ signals (Eq. 41) differ significantly from the simplified one (Eq. 27) only at sufficiently large time $t\geq \tau_{\mathrm{DQ}}^{\mathrm{fl}}$ , where $\tau_{\mathrm{DQ}}^{\mathrm{fl}}$ is the characteristic time of the flip-flop processes determined by the value ${\tilde{P}}_{ij}^{n,\mathrm{fl}}\left\{t;0\right\}$ (see Eq. 38). Now we will try to express this time through the effective spin–spin relaxation time, $T_{2}^{\mathrm{eff}}$ , defined by the relation $g^{\mathrm{FID}}\left(T_{2}^{\mathrm{eff}}\right)=e^{-1}$ , where $g^{\mathrm{FID}}\left(t\right)$ is the FID or Hahn echo signal governed by dipolar dephasing and an approximately Gaussian initial decay. To do this, let us begin by analyzing the modified Anderson–Weiss approximation for $g^{\mathrm{FID}}\left(t\right)$ obtained in Fatkullin et al. (2012) and written in terms of the notation of this paper:

65 $$\begin{array}{rl} g^{\mathrm{FID}}\left(t\right) & =\mathrm{exp}\{-\frac{3}{4}I\left(I+1\right)\frac{4}{N_{s}}\sum_{k,l}{}^{\prime }\int_{0}^{t}\\ & d\tau \left(t-\tau \right){\langle \omega_{kl}\left(\tau \right)\omega_{kl}\left(0\right)\rangle }_{\mathrm{eq}}P_{kl}^{\mathrm{FID},\mathrm{fl}}\left(\tau \right)\}. \end{array}$$

Since we are interested in relatively short times in the relation (64), we can set $P_{kl}^{\mathrm{FID},\mathrm{fl}}\left(\tau \right)=1$ , which, combined with the assumption that all spins have the same environment, allows us to convert it to the form

66 $$g^{\mathrm{FID}}\left(t\right)=\mathrm{exp}\left\{-\frac{3}{2}I\left(I+1\right)\sum_{l}{}^{\prime }\left({\langle {\left(\varphi_{kl}^{\left(0\right)}\left(t;0\right)\right)}^{2}\rangle }_{\mathrm{eq}}\right)\right\}.$$

If we apply the Anderson–Weiss approximation to Eq. (45), we obtain

67 $$\begin{array}{rl} P_{ij}^{n,\mathrm{fl}}\left\{t;0\right\} & =\mathrm{exp}\{-\frac{1}{6}I\left(I+1\right)\sum_{k}{}^{\prime }({\langle {\left(\varphi_{ik}^{\left(n\right)}\left(t;0\right)\right)}^{2}\rangle }_{\mathrm{eq}}\\ & +{\langle {\left(\varphi_{jk}^{\left(n\right)}\left(t;0\right)\right)}^{2}\rangle }_{\mathrm{eq}})\}\\ & =\mathrm{exp}\{-\frac{1}{3}I\left(I+1\right)\sum_{k}{}^{\prime }\left({\langle {\left(\varphi_{ik}^{\left(n\right)}\left(t;0\right)\right)}^{2}\rangle }_{\mathrm{eq}}\right)\}, \end{array}$$

where we determine that $\sum_{k}{}^{\prime }{\langle {\left(\varphi_{ik}^{\left(n\right)}\left(t;0\right)\right)}^{2}\rangle }_{\mathrm{eq}}=\sum_{k}{}^{\prime }{\langle {\left(\varphi_{jk}^{\left(n\right)}\left(t;0\right)\right)}^{2}\rangle }_{\mathrm{eq}}$ . Note that by virtue of the last approximation, the values of $P_{ij}^{0,\mathrm{fl}}\left\{t;0\right\}$ and $P_{k}^{\mathrm{FID},\mathrm{fl}}\left(t\right)$ are equal for n = 0.

Let us now consider the case of the DQ experiment, n = 0; i.e., no time reversal operation with respect to spin variables is performed at time $t=\tau_{\mathrm{DQ}}$ . We see that the expressions for $g^{\mathrm{FID}}\left(t\right)$ and $P_{ij}^{0,\mathrm{fl}}\left\{t;0\right\}$ are similar. Now we can determine the corresponding characteristic times by means of the relations:

68 $$\begin{array}{rl} & g^{\mathrm{FID}}\left(t\right)=\mathrm{exp}\left\{-{\left(\frac{t}{T_{2}^{\mathrm{eff}}}\right)}^{\alpha }\right\},\\ & P_{ij}^{0,\mathrm{fl}}\left\{t;0\right\}=\mathrm{exp}\left\{-{\left(\frac{t}{T_{ij}^{\mathrm{DQ},\mathrm{fl}}}\right)}^{\alpha }\right\}, \end{array}$$

where 1≤α≤2 is the system-dependent exponent. In solids, α=2 ; in high molecular polymer melts, 1.25≤α≤1.75 (see, for example, Kimmich and Fatkullin, 2017; Rössler et al., 2013; Fatkullin et al., 2015); and for low molecular liquids, α=1 (see, for example, Mehring, 1983; Abragam, 1961). From Eqs. (66), (67) and (68) we deduce the following relation between the discussed characteristic times:

69 $$T_{ij}^{\mathrm{DQ},\mathrm{fl}}={\left(\frac{9}{2}\right)}^{1/\alpha }T_{2}^{\mathrm{eff}}.$$

We see that the numerical coefficient linking the two characteristic times is quite large even with the largest possible value of the exponent:

70 $$\alpha =2:T_{ij}^{\mathrm{DQ},\mathrm{fl}}={\left(\frac{9}{2}\right)}^{1/2}T_{2}^{\mathrm{eff}}\approx 2.12T_{2}^{\mathrm{eff}}.$$

The latter suggests considering the time ${\left(\frac{9}{2}\right)}^{1/2}T_{2}^{\mathrm{eff}}\approx 2.12T_{2}^{\mathrm{eff}}$ as a lower bound starting from which the influence of flip-flop processes becomes dominant. The duration of the DQ experiment is equal to $2\tau_{\mathrm{DQ}}$ , so the influence of intermolecular flip-flop processes on the experimentally measured signal can be neglected at times $\tau_{\mathrm{DQ}}<{\left(\frac{9}{8}\right)}^{1/2}T_{2}^{\mathrm{eff}}\approx 1.06T_{2}^{\mathrm{eff}}$ .

## 3 Discussion

### 3.1 Effect of flip-flop processes

Relations (41)–(44) are the main general results of this paper. They have a formal mathematical structure with a more simplified approach (see relation 27), completely neglecting flip-flops during DQ NMR experiments. The difference is hidden in the values $\varphi_{ij}^{\left(n\right)}=\int_{0}^{2\tau_{\mathrm{DQ}}}\omega_{ij}^{\left(n\right)}\left(t_{1}\right)dt_{1}$ in Eq. (27) and ${\tilde{\varphi }}_{ij}^{\left(n\right)}=\int_{0}^{2\tau_{\mathrm{DQ}}}{\tilde{\omega }}_{ij}^{\left(n\right)}\left(t_{1}\right)dt_{1}$ , with ${\tilde{\omega }}_{ij}^{\left(n\right)}\left(t\right)={\tilde{P}}_{ij}^{n,\mathrm{fl}}\left(t;0\right)\omega_{ij}^{\left(n\right)}\left(t\right)$ , in Eqs. (41)–(44). As we can see, the influence of flip-flop processes was taken into account by multiplying each frequency $\omega_{ij}^{\left(n\right)}\left(t_{1}\right)$ by the value of ${\tilde{P}}_{ij}^{n,\mathrm{fl}}\left(t;0\right)$ , defined by Eq. (32). As it is argued in Sect. 2.4, this quantity is closely related to the probabilities of spins with numbers i and j during the time interval t to not participate in flip-flop processes with any other spin of the system. This quantity is a complex function of the lattice variables. However, after averaging over the lattice variable $P_{ij}^{n,\mathrm{fl}}\left\{t_{2};t_{1}\right\}\equiv {\langle {\tilde{P}}_{ij}^{n,\mathrm{fl}}\left\{t_{2};t_{1}\right\}\rangle }_{\mathrm{eq}}={\langle {\tilde{P}}_{ij}^{n,\mathrm{fl}}\left\{t_{2}-t_{1};0\right\}\rangle }_{\mathrm{eq}}$ (see relation 45), it provides the probability that the spins with numbers i and j have their initial time-zero mutual orientation at time $t_{2}-t_{1}$ . For times shorter than the characteristic flip-flop transition time $\left|t_{2}-t_{1}\right|\ll T_{ij}^{DQ,\mathrm{fl}}$ , this means that the discussed spins did not undergo flip-flop transitions with any other spins in the system. For the case of a (quasi-)rigid lattice, our approach allows us to obtain the reversibility of the DQ experiment in time for the case n = 1 (see Eqs. 50 and 51) and after using an Anderson–Weiss-type approximation for the transition from Eqs. (58) to (59). Experimentally measurable normalized buildup functions have a rather simple mathematical structure as represented by Eq. (60).

### 3.2 Numerical results and comparison with spin dynamics simulations

We now turn to comparing the results of Sect. 2.5, specifically the quasi-static approximation, to results of spin dynamics simulations based upon solving the Liouville–von Neumann equation in small time steps for finite few-spin systems for the explicit BP pulse sequence (as well as simple FIDs after a 90° pulse), while always assuming δ pulses. For time efficiency, we did not simulate the two different BP experiments with variable reconversion phase but using a fixed 90° phase shift and filtering the density matrix for DQ coherences after the excitation block, thus directly calculating the DQ buildup curve. We used an earlier home-written code (Saalwächter and Fischbach, 2002) that is not optimized (no sparse-matrix algebra is implemented), which means that simulations are limited to eight spins due to the large dimension (up to 2^8^) of the density matrix and the operators/propagators. We implemented the analytical solution, Eqs. (60) and (61), on the basis of the very same code, using the same core routines handling the dipolar interaction tensors and the same input files with spin system parameters.

**Figure 1:**
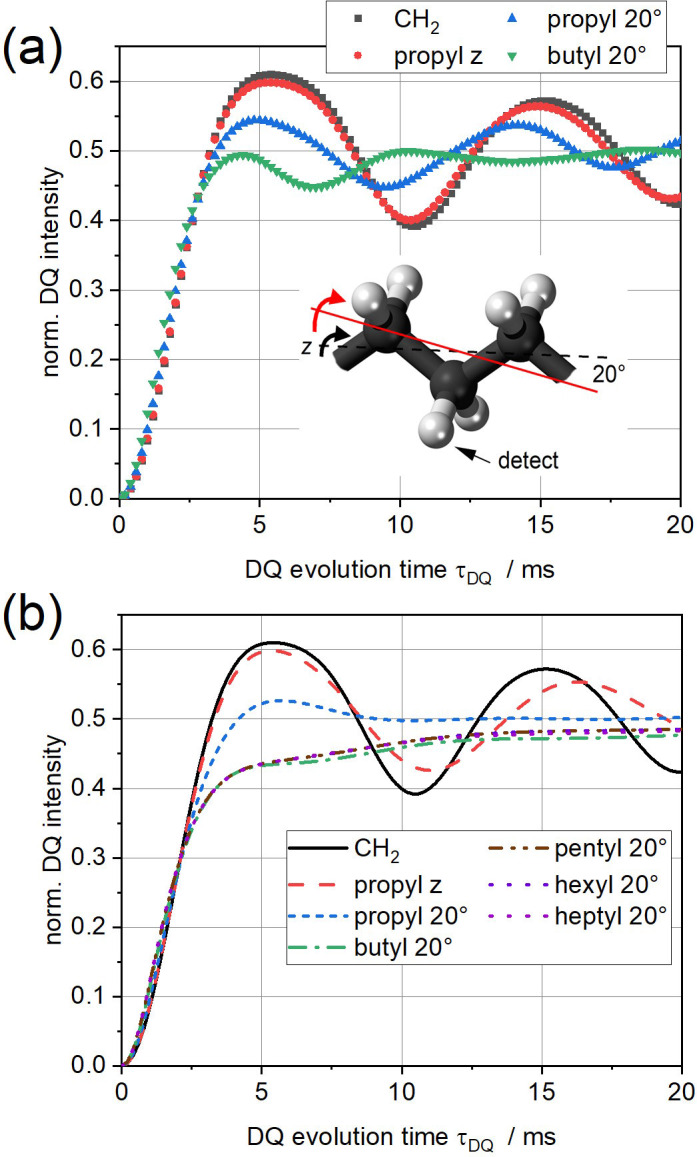
^1^H DQ buildup curves of all-trans alkyl cutouts rotating about the molecular long axis ( z ) or about an axis inclined by 20° **(a)** from spin dynamics simulations and **(b)** analytically calculated from Eqs. (60) and (61).

As to spin systems, we aimed at mimicking a simple main-chain protonated polymer, where the chain motions provide a fast-limit average of all conformations between two cross-links or entanglements. This provides uniaxial averaging of all intra-chain dipolar tensors, resulting in residual coupling tensors that are all colinear (parallel to the end-to-end distance of the chain) and reduced in magnitude by a factor of about 100 compared to the static limit (Saalwächter, 2007). A physically realistic model would have to be based on a trajectory of a molecular dynamics simulation. For simplicity, we chose to simulate cutouts of all-trans alkane structures (CH ${}_{2}{)}_{n}$ using canonical CH and CC distances of 0.109 and 0.154 nm, respectively, assuming tetrahedral symmetry (see the inset of Fig. 1a). This model provides $r_{\mathrm{HH}}=0.178$ nm and thus an intra-CH_2_ static-limit coupling constant of $D^{\mathrm{HH}}/2\pi =24$ Hz (see Eq. 2). We always detect (or calculate for) the central protons. Uniaxial averaging is implemented by symmetric three-site jumps mimicking fast rigid-body rotation (leading to a scaling of the HH dipolar couplings by - 0.5 when the HH bond is perpendicular to the rotation axis), providing a situation with all-colinear dipolar tensors. To reach residual couplings corresponding to those of polymer melts, a scaling factor of 0.01 was applied to all couplings, leading to a dominant intra-CH_2_ residual dipolar coupling constant, $D_{\mathrm{res}}^{\mathrm{HH}}/2\pi =122$ Hz. Another set of simulations considered a propyl fragment in $g^{+}g^{+}$ conformation (locating two outer protons in the CCC plane) with up to two additional protons located at the van der Waals distance above either of the two central protons (with an unscaled remote coupling of $D_{\mathrm{HH}}/2\pi \approx 9$ kHz). Powder averaging was performed over only 40 angles of β between the main axis of the averaged tensor and the magnetic field (as we simulate in the time domain, convergence was reached within the discussed limited time intervals).

Simulation results of DQ buildup curves are compared in Fig. 1a for a CH_2_ group (for which simulation and analytical prediction are identical) and for rotating alkyl cutouts starting with propyl (six protons). For rotation around the all-trans ( z ) axis, the secondary couplings of the central CH_2_ protons to the ones on the side are very small after uniaxial z averaging, due to angles between the HH vectors and the z axis being close to the magic angle. This is obvious from the very small difference between the CH_2_ and the “propyl z “ responses. To mimic a more complex spin system with a larger spread of couplings, we inclined the rotation axis by 20°, rending the couplings of the central CH_2_ group to the ones on the different sides different. As a result, the coherent oscillations are significantly damped. Adding more CH_2_ groups (with butyl being the largest feasible spin system for the simulations) damps the oscillations even more, leading to a buildup curve that reaches the expected plateau at $I_{\mathrm{DQ}}=0.5$ from below.

**Figure 2:**
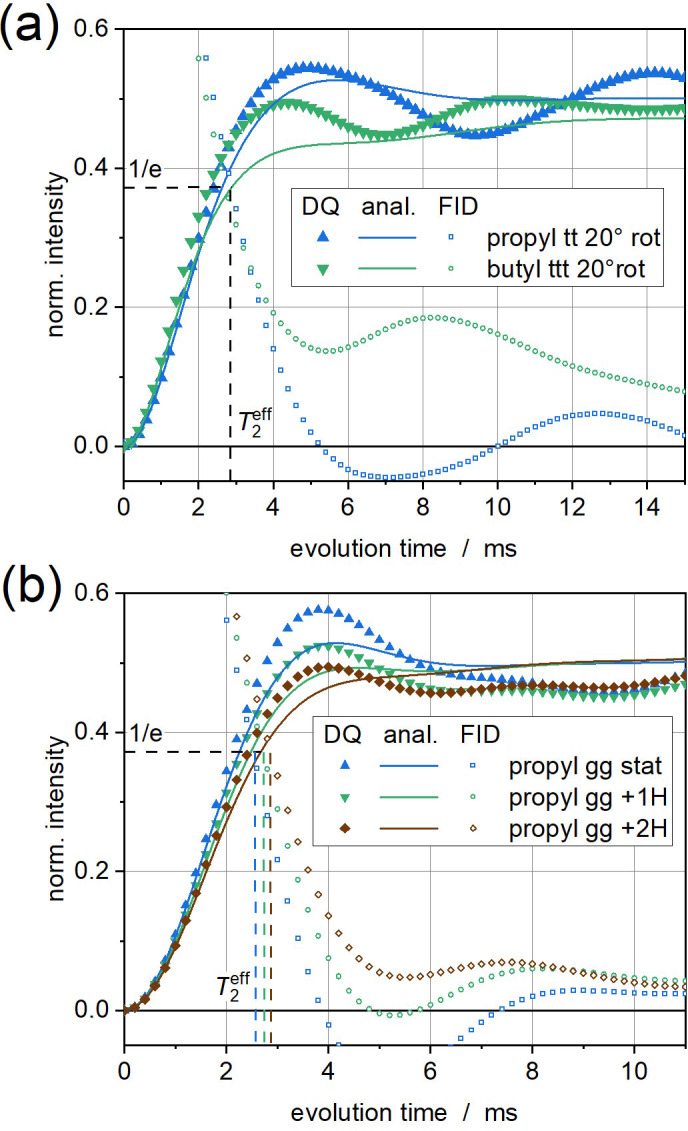
^1^H DQ buildup curves of alkyl cutouts, comparing spin dynamics simulation results and analytical calculations, **(a)** in all-trans conformation rotating about an axis inclined by 20° (all couplings scaled by 0.01; see Fig. 1) and **(b)** propyl (six spins) in static $g^{+}g^{+}$ conformation (all couplings scaled by 0.005), with up to two additional remote protons located at the van der Waals distance above either of the two central CH_2_ protons ( $D_{\mathrm{HH}}/2\pi \approx 9$ kHz). Simulated FID signals are also shown to indicate $T_{2}^{\mathrm{eff}}$ .

The analytical results shown in Fig. 1b, for the first time possible for systems beyond a spin pair, mimic these trends surprisingly well. Notably, the changes in the buildup curves upon adding more CH_2_ groups, which is easily possible in the analytical calculations, does not change the result significantly. For a more quantitative comparison, we directly compare in Fig. 2a the simulations and analytical calculations for the propyl 20° and butyl 20° cases. While the agreement between the former pair is very satisfactory, large deviations are observed in the latter case. This may well be due to the finite spin system and specificities related to the all-colinear dipolar tensors. In Fig. 2, we also plot simulated FIDs, which can be used to extract $T_{2}^{\mathrm{eff}}$ . Up to $\tau_{\mathrm{DQ}}=T_{2}^{\mathrm{eff}}$ , simulated and analytical results match within 15 %. To explore the effect of the all-tensor colinearity in these calculations, we add in Fig. 2b simulations and calculations for a static three-dimensional spin system (with a dipolar scaling factor of 0.005 to arrive at a similar coupling magnitude as before), in this case of propyl fragment in $g^{+}g^{+}$ conformation, optionally adding two remote protons. It is observed that the agreement between simulations and analytical solutions within $T_{2}^{\mathrm{eff}}$ is generally even better, confirming the hypothesis that a three-dimensional distribution of variable coupling tensors is maybe a better basis for the application of the AW approximation inherent to Eq. (67).

Thus, for cases when we have a large scatter in the coupling constants for different spins, this result, in our opinion, can be considered quite satisfactory. The improvement of the result requires a more detailed treatment of flip-flop processes between different spins than we have done in the transition from relation (45) to relation (46), which does not take into account the returns of spin polarization during spin diffusion to the initial spin, which will lead to a slower decay of the function in Eq. (46) at time $\tau_{\mathrm{DQ}}\geq T_{2}^{\mathrm{eff}}$ . Also at the discussed times, it will become necessary to improve the approximation in Eq. (32) due to the simultaneous exchange of two different pairs of spins by their mutual spin polarizations (see the remark after Eq. 37). It may also be important to further develop the ideas presented by Bochkin et al. (2022, 2024) and Fel'dman et al. (2022).

As a final note, a more detailed assessment, using more realistic and much larger spin systems, was beyond the scope of the present work. With larger spin systems as well as more current simulation software and increased computing power, the agreement between simulations and theory on the one hand and experimental results on the other hand is of course expected to be even better. Here, we note that our analytical approximation, so far valid up to $\tau_{\mathrm{DQ}}=T_{2}^{\mathrm{eff}}$ , will need to be improved by consideration of higher-order corrections. On the other hand, our simplistic approach to setting up the spin systems is not expected to provide any better agreement as long as the local conformational dynamics, and also intermolecular couplings are not realistically considered. This will require the combination of spin dynamics simulations with (in the simplest case) pre-averaged interaction tensors extracted from trajectories of atomistic molecular dynamics simulations.

## 4 Conclusions

The mathematical identity, Eq. (7), allows us to reformulate the derivation of experimentally measured signals in DQ NMR experiments in such a way that taking into account the effects of inter-spin flip-flop processes is natural and simple. In this way, it was possible for the first time to provide an analytical calculation of DQ buildup curves in multi-spin systems. From a formal point of view, it all comes down to redefining the phases of mutual rotations of spins induced by the DQ Hamiltonian Eq. (4); cf. relations (27) and (41), (44). The influence of flip-flop transitions translates to phases that are linearly dependent on the conditional probability ${\tilde{P}}_{ij}^{n,\mathrm{fl}}\left(t\right)$ that the corresponding pair of spins did not participate in flip-flops with any other spin of the system during the time interval t ; see Eqs. (40) and (42). The structure of the DQ Hamiltonian Eq. (4) itself is such that the inherent flip-flop probabilities are half the size of those induced by the secular part of the dipole–dipole interaction Hamiltonian Eq. (1). The latter allows us to neglect the effects of flip-flop processes in DQ experiments and use the simplified description given by relation (27) of sufficiently long timescales in units of the effective spin–spin relaxation time, $t<2.12T_{2}^{\mathrm{eff}}$ . A comparison of the predictions with spin dynamics simulations of simple, small spin systems of different sizes provided a promising, near-quantitative agreement for $\tau_{\mathrm{DQ}}\leq 1.06T_{2}^{\mathrm{eff}}$ , yet the origin of existing deviations for longer times requires further work.

## Data Availability

The simulation codes as well as datasets generated and analyzed for this study as they appear in the figures of this article can be found on the Zenodo repository (10.5281/zenodo.13628349, Saalwächter and Fatkullin, 2024).
